# Supplementary material for: Secretory Profile Analysis of Human Granulosa Cell Line Following Gonadotropin Stimulation
Source: Int J Mol Sci. 2025 Apr 25;26(9):4108. doi: 10.3390/ijms26094108 (PMC12072160; doi:10.3390/ijms26094108)
Supplement: Supplementary file 1 [file ijms-26-04108-s001.zip › Table S3-Path hCG_FSH+hCG based on FC.docx]

**PATHWAYS ANALYSIS BASED ON FOLD CHANGE (hCG/ FSH+hCG)**

**First 15 pathways based on 8 Up-regulated proteins**

| Pathway identifier | Pathway name | #Entities found | #Entities total | Entities pValue | Entities FDR |
| --- | --- | --- | --- | --- | --- |
| R-HSA-5652227 | Fructose biosynthesis | 1 | 2 | 1.38E-03 | 4.11E-02 |
| R-HSA-114608 | Platelet degranulation | 2 | 128 | 3.25E-03 | 4.11E-02 |
| R-HSA-8964026 | Chylomicron clearance | 1 | 5 | 3.44E-03 | 4.11E-02 |
| R-HSA-76005 | Response to elevated platelet cytosolic Ca2+ | 2 | 133 | 3.50E-03 | 4.11E-02 |
| R-HSA-5652084 | Fructose metabolism | 1 | 7 | 4.81E-03 | 4.11E-02 |
| R-HSA-159763 | Transport of gamma-carboxylated protein precursors from the endoplasmic reticulum to the Golgi apparatus | 1 | 9 | 6.18E-03 | 4.11E-02 |
| R-HSA-390450 | Folding of actin by CCT/TriC | 1 | 10 | 6.86E-03 | 4.11E-02 |
| R-HSA-159782 | Removal of aminoterminal propeptides from gamma-carboxylated proteins | 1 | 10 | 6.86E-03 | 4.11E-02 |
| R-HSA-159740 | Gamma-carboxylation of protein precursors | 1 | 10 | 6.86E-03 | 4.11E-02 |
| R-HSA-159854 | Gamma-carboxylation, transport, and amino-terminal cleavage of proteins | 1 | 11 | 7.55E-03 | 4.11E-02 |
| R-HSA-196108 | Pregnenolone biosynthesis | 1 | 12 | 8.23E-03 | 4.11E-02 |
| R-HSA-8964038 | LDL clearance | 1 | 19 | 1.30E-02 | 4.72E-02 |
| R-HSA-76002 | Platelet activation, signaling and aggregation | 2 | 265 | 1.33E-02 | 4.72E-02 |
| R-HSA-140875 | Common Pathway of Fibrin Clot Formation | 1 | 22 | 1.50E-02 | 4.72E-02 |
| R-HSA-9013418 | RHOBTB2 GTPase cycle | 1 | 23 | 1.57E-02 | 4.72E-02 |

| Fructose biosynthesis | |
| --- | --- |
| P15121 | Aldo-keto reductase family 1 member B1 OS=Homo sapiens OX=9606 GN=AKR1B1 PE=1 SV=3 |

| Platelet degranulation | |
| --- | --- |
| Q92520 | Protein FAM3C OS=Homo sapiens OX=9606 GN=FAM3C PE=1 SV=1 |
| P07225 | Vitamin K-dependent protein S OS=Homo sapiens OX=9606 GN=PROS1 PE=1 SV=1 |

| Chylomicron clearance | |
| --- | --- |
| P01130 | Low-density lipoprotein receptor OS=Homo sapiens OX=9606 GN=LDLR PE=1 SV=1 |

| Response to elevated platelet cytosolic Ca2+ | |
| --- | --- |
| Q92520 | Protein FAM3C OS=Homo sapiens OX=9606 GN=FAM3C PE=1 SV=1 |
| P07225 | Vitamin K-dependent protein S OS=Homo sapiens OX=9606 GN=PROS1 PE=1 SV=1 |

| Fructose metabolism | |
| --- | --- |
| P15121 | Aldo-keto reductase family 1 member B1 OS=Homo sapiens OX=9606 GN=AKR1B1 PE=1 SV=3 |

| Transport of gamma-carboxylated protein precursors from the endoplasmic reticulum to the Golgi apparatus | |
| --- | --- |
| P07225 | Vitamin K-dependent protein S OS=Homo sapiens OX=9606 GN=PROS1 PE=1 SV=1 |

| Folding of actin by CCT/TriC | |
| --- | --- |
| P40227 | T-complex protein 1 subunit zeta OS=Homo sapiens OX=9606 GN=CCT6A PE=1 SV=3 |

| Removal of aminoterminal propeptides from gamma-carboxylated proteins | |
| --- | --- |
| P07225 | Vitamin K-dependent protein S OS=Homo sapiens OX=9606 GN=PROS1 PE=1 SV=1 |

| Gamma-carboxylation of protein precursors | |
| --- | --- |
| P07225 | Vitamin K-dependent protein S OS=Homo sapiens OX=9606 GN=PROS1 PE=1 SV=1 |

| Gamma-carboxylation, transport, and amino-terminal cleavage of proteins | |
| --- | --- |
| P07225 | Vitamin K-dependent protein S OS=Homo sapiens OX=9606 GN=PROS1 PE=1 SV=1 |

| Pregnenolone biosynthesis | |
| --- | --- |
| P15121 | Aldo-keto reductase family 1 member B1 OS=Homo sapiens OX=9606 GN=AKR1B1 PE=1 SV=3 |

| LDL clearance | |
| --- | --- |
| P01130 | Low-density lipoprotein receptor OS=Homo sapiens OX=9606 GN=LDLR PE=1 SV=1 |

| Platelet activation, signaling and aggregation | |
| --- | --- |
| Q92520 | Protein FAM3C OS=Homo sapiens OX=9606 GN=FAM3C PE=1 SV=1 |
| P07225 | Vitamin K-dependent protein S OS=Homo sapiens OX=9606 GN=PROS1 PE=1 SV=1 |

| Common Pathway of Fibrin Clot Formation | |
| --- | --- |
| P07225 | Vitamin K-dependent protein S OS=Homo sapiens OX=9606 GN=PROS1 PE=1 SV=1 |

| RHOBTB2 GTPase cycle | |
| --- | --- |
| P40227 | T-complex protein 1 subunit zeta OS=Homo sapiens OX=9606 GN=CCT6A PE=1 SV=3 |

**First 15 pathways based on 12 Up-regulated proteins**

| Pathway identifier | Pathway name | #Entities found | #Entities total | Entities pValue | Entities FDR |
| --- | --- | --- | --- | --- | --- |
| R-HSA-3299685 | Detoxification of Reactive Oxygen Species | 2 | 39 | 7.27E-04 | 7.49E-02 |
| R-HSA-6798695 | Neutrophil degranulation | 4 | 478 | 1.08E-03 | 7.49E-02 |
| R-HSA-5218859 | Regulated Necrosis | 2 | 62 | 1.81E-03 | 8.34E-02 |
| R-HSA-1266738 | Developmental Biology | 5 | 1162 | 4.32E-03 | 9.36E-02 |
| R-HSA-2262752 | Cellular responses to stress | 4 | 769 | 6.16E-03 | 9.36E-02 |
| R-HSA-111459 | Activation of caspases through apoptosome-mediated cleavage | 1 | 6 | 6.18E-03 | 9.36E-02 |
| R-HSA-8953897 | Cellular responses to stimuli | 4 | 783 | 6.57E-03 | 9.36E-02 |
| R-HSA-111464 | SMAC(DIABLO)-mediated dissociation of IAP:caspase complexes | 1 | 7 | 7.20E-03 | 9.36E-02 |
| R-HSA-111463 | SMAC (DIABLO) binds to IAPs | 1 | 7 | 7.20E-03 | 9.36E-02 |
| R-HSA-111457 | Release of apoptotic factors from the mitochondria | 1 | 7 | 7.20E-03 | 9.36E-02 |
| R-HSA-111469 | SMAC, XIAP-regulated apoptotic response | 1 | 8 | 8.23E-03 | 9.87E-02 |
| R-HSA-111458 | Formation of apoptosome | 1 | 11 | 1.13E-02 | 1.01E-01 |
| R-HSA-9627069 | Regulation of the apoptosome activity | 1 | 11 | 1.13E-02 | 1.01E-01 |
| R-HSA-111461 | Cytochrome c-mediated apoptotic response | 1 | 13 | 1.33E-02 | 1.01E-01 |
| R-HSA-5210891 | Uptake and function of anthrax toxins | 1 | 16 | 1.64E-02 | 1.01E-01 |

| Detoxification of Reactive Oxygen Species | |
| --- | --- |
| P99999 | Cytochrome c OS=Homo sapiens OX=9606 GN=CYCS PE=1 SV=2 |
| P30041 | Peroxiredoxin-6 OS=Homo sapiens OX=9606 GN=PRDX6 PE=1 SV=3 |

| Neutrophil degranulation | |
| --- | --- |
| P61160 | Actin-related protein 2 OS=Homo sapiens OX=9606 GN=ACTR2 PE=1 SV=1 |
| P30041 | Peroxiredoxin-6 OS=Homo sapiens OX=9606 GN=PRDX6 PE=1 SV=3 |
| P04264 | Keratin, type II cytoskeletal 1 OS=Homo sapiens OX=9606 GN=KRT1 PE=1 SV=6 |
| Q92820 | Gamma-glutamyl hydrolase OS=Homo sapiens OX=9606 GN=GGH PE=1 SV=2 |

| Regulated Necrosis | |
| --- | --- |
| P99999 | Cytochrome c OS=Homo sapiens OX=9606 GN=CYCS PE=1 SV=2 |
| Q8WUM4 | Programmed cell death 6-interacting protein OS=Homo sapiens OX=9606 GN=PDCD6IP PE=1 SV=1 |

| Developmental Biology | |
| --- | --- |
| P61160 | Actin-related protein 2 OS=Homo sapiens OX=9606 GN=ACTR2 PE=1 SV=1 |
| O75326 | Semaphorin-7A OS=Homo sapiens OX=9606 GN=SEMA7A PE=1 SV=1 |
| P30050 | 60S ribosomal protein L12 OS=Homo sapiens OX=9606 GN=RPL12 PE=1 SV=1 |
| Q16576 | Histone-binding protein RBBP7 OS=Homo sapiens OX=9606 GN=RBBP7 PE=1 SV=1 |
| P04264 | Keratin, type II cytoskeletal 1 OS=Homo sapiens OX=9606 GN=KRT1 PE=1 SV=6 |

| Cellular responses to stress | |
| --- | --- |
| P30050 | 60S ribosomal protein L12 OS=Homo sapiens OX=9606 GN=RPL12 PE=1 SV=1 |
| P30041 | Peroxiredoxin-6 OS=Homo sapiens OX=9606 GN=PRDX6 PE=1 SV=3 |
| P99999 | Cytochrome c OS=Homo sapiens OX=9606 GN=CYCS PE=1 SV=2 |
| Q16576 | Histone-binding protein RBBP7 OS=Homo sapiens OX=9606 GN=RBBP7 PE=1 SV=1 |

| Activation of caspases through apoptosome-mediated cleavage | |
| --- | --- |
| P99999 | Cytochrome c OS=Homo sapiens OX=9606 GN=CYCS PE=1 SV=2 |

| Cellular responses to stimuli | |
| --- | --- |
| P30050 | 60S ribosomal protein L12 OS=Homo sapiens OX=9606 GN=RPL12 PE=1 SV=1 |
| P30041 | Peroxiredoxin-6 OS=Homo sapiens OX=9606 GN=PRDX6 PE=1 SV=3 |
| P99999 | Cytochrome c OS=Homo sapiens OX=9606 GN=CYCS PE=1 SV=2 |
| Q16576 | Histone-binding protein RBBP7 OS=Homo sapiens OX=9606 GN=RBBP7 PE=1 SV=1 |

| SMAC(DIABLO)-mediated dissociation of IAP:caspase complexes | |
| --- | --- |
| P99999 | Cytochrome c OS=Homo sapiens OX=9606 GN=CYCS PE=1 SV=2 |

| SMAC (DIABLO) binds to IAPs | |
| --- | --- |
| P99999 | Cytochrome c OS=Homo sapiens OX=9606 GN=CYCS PE=1 SV=2 |

| Release of apoptotic factors from the mitochondria | |
| --- | --- |
| P99999 | Cytochrome c OS=Homo sapiens OX=9606 GN=CYCS PE=1 SV=2 |

| SMAC, XIAP-regulated apoptotic response | |
| --- | --- |
| P99999 | Cytochrome c OS=Homo sapiens OX=9606 GN=CYCS PE=1 SV=2 |

| Formation of apoptosome | |
| --- | --- |
| P99999 | Cytochrome c OS=Homo sapiens OX=9606 GN=CYCS PE=1 SV=2 |

| Regulation of the apoptosome activity | |
| --- | --- |
| P99999 | Cytochrome c OS=Homo sapiens OX=9606 GN=CYCS PE=1 SV=2 |

| Cytochrome c-mediated apoptotic response | |
| --- | --- |
| P99999 | Cytochrome c OS=Homo sapiens OX=9606 GN=CYCS PE=1 SV=2 |

| Uptake and function of anthrax toxins | |
| --- | --- |
| Q8WUM4 | Programmed cell death 6-interacting protein OS=Homo sapiens OX=9606 GN=PDCD6IP PE=1 SV=1 |
