# Supplementary material for: Secretory Profile Analysis of Human Granulosa Cell Line Following Gonadotropin Stimulation
Source: Int J Mol Sci. 2025 Apr 25;26(9):4108. doi: 10.3390/ijms26094108 (PMC12072160; doi:10.3390/ijms26094108)
Supplement: Supplementary file 1 [file ijms-26-04108-s001.zip › Table S2-Path FSH_FSH+hCG based on FC.docx]

**PATHWAYS ANALYSIS BASED ON FOLD CHANGE (FSH/FSH+hCG)**

**First 15 pathways based on 39 Up-regulated proteins**

| Pathway identifier | Pathway name | #Entities found | #Entities total | Entities pValue | Entities FDR |
| --- | --- | --- | --- | --- | --- |
| R-HSA-3000171 | Non-integrin membrane-ECM interactions | 7 | 59 | 1.40E-09 | 1.71E-07 |
| R-HSA-1474244 | Extracellular matrix organization | 11 | 300 | 3.92E-09 | 2.39E-07 |
| R-HSA-3000178 | ECM proteoglycans | 7 | 76 | 7.89E-09 | 3.15E-07 |
| R-HSA-1474228 | Degradation of the extracellular matrix | 8 | 140 | 2.42E-08 | 7.25E-07 |
| R-HSA-3000157 | Laminin interactions | 5 | 30 | 6.99E-08 | 1.40E-06 |
| R-HSA-8874081 | MET activates PTK2 signaling | 5 | 30 | 6.99E-08 | 1.40E-06 |
| R-HSA-8875878 | MET promotes cell motility | 5 | 41 | 3.24E-07 | 5.51E-06 |
| R-HSA-8957275 | Post-translational protein phosphorylation | 6 | 107 | 1.79E-06 | 2.68E-05 |
| R-HSA-2022090 | Assembly of collagen fibrils and other multimeric structures | 5 | 61 | 2.25E-06 | 2.92E-05 |
| R-HSA-1650814 | Collagen biosynthesis and modifying enzymes | 5 | 67 | 3.54E-06 | 4.25E-05 |
| R-HSA-381426 | Regulation of Insulin-like Growth Factor (IGF) transport and uptake by Insulin-like Growth Factor Binding Proteins (IGFBPs) | 6 | 124 | 4.15E-06 | 4.56E-05 |
| R-HSA-6806834 | Signaling by MET | 5 | 80 | 8.32E-06 | 8.32E-05 |
| R-HSA-216083 | Integrin cell surface interactions | 5 | 85 | 1.11E-05 | 1.00E-04 |
| R-HSA-419037 | NCAM1 interactions | 4 | 42 | 1.40E-05 | 1.12E-04 |
| R-HSA-1474290 | Collagen formation | 5 | 90 | 1.46E-05 | 1.17E-04 |

| Non-integrin membrane-ECM interactions | |
| --- | --- |
| P02462 | Collagen alpha-1(IV) chain OS=Homo sapiens OX=9606 GN=COL4A1 PE=1 SV=4 |
| P02461 | Collagen alpha-1(III) chain OS=Homo sapiens OX=9606 GN=COL3A1 PE=1 SV=4 |
| P55268 | Laminin subunit beta-2 OS=Homo sapiens OX=9606 GN=LAMB2 PE=1 SV=2 |
| P05997 | Collagen alpha-2(V) chain OS=Homo sapiens OX=9606 GN=COL5A2 PE=1 SV=3 |
| O15230 | Laminin subunit alpha-5 OS=Homo sapiens OX=9606 GN=LAMA5 PE=1 SV=8 |
| P20908 | Collagen alpha-1(V) chain OS=Homo sapiens OX=9606 GN=COL5A1 PE=1 SV=3 |
| P98160 | Basement membrane-specific heparan sulfate proteoglycan core protein OS=Homo sapiens OX=9606 GN=HSPG2 PE=1 SV=4 |

| Extracellular matrix organization | |
| --- | --- |
| P02462 | Collagen alpha-1(IV) chain OS=Homo sapiens OX=9606 GN=COL4A1 PE=1 SV=4 |
| P02461 | Collagen alpha-1(III) chain OS=Homo sapiens OX=9606 GN=COL3A1 PE=1 SV=4 |
| P55268 | Laminin subunit beta-2 OS=Homo sapiens OX=9606 GN=LAMB2 PE=1 SV=2 |
| P05997 | Collagen alpha-2(V) chain OS=Homo sapiens OX=9606 GN=COL5A2 PE=1 SV=3 |
| O95967 | EGF-containing fibulin-like extracellular matrix protein 2 OS=Homo sapiens OX=9606 GN=EFEMP2 PE=1 SV=3 |
| O15230 | Laminin subunit alpha-5 OS=Homo sapiens OX=9606 GN=LAMA5 PE=1 SV=8 |
| P20908 | Collagen alpha-1(V) chain OS=Homo sapiens OX=9606 GN=COL5A1 PE=1 SV=3 |
| P98095 | Fibulin-2 OS=Homo sapiens OX=9606 GN=FBLN2 PE=1 SV=2 |
| P98160 | Basement membrane-specific heparan sulfate proteoglycan core protein OS=Homo sapiens OX=9606 GN=HSPG2 PE=1 SV=4 |
| P13497 | Bone morphogenetic protein 1 OS=Homo sapiens OX=9606 GN=BMP1 PE=1 SV=2 |
| P14543 | Nidogen-1 OS=Homo sapiens OX=9606 GN=NID1 PE=1 SV=3 |

| ECM proteoglycans | |
| --- | --- |
| P02462 | Collagen alpha-1(IV) chain OS=Homo sapiens OX=9606 GN=COL4A1 PE=1 SV=4 |
| P02461 | Collagen alpha-1(III) chain OS=Homo sapiens OX=9606 GN=COL3A1 PE=1 SV=4 |
| P55268 | Laminin subunit beta-2 OS=Homo sapiens OX=9606 GN=LAMB2 PE=1 SV=2 |
| P05997 | Collagen alpha-2(V) chain OS=Homo sapiens OX=9606 GN=COL5A2 PE=1 SV=3 |
| O15230 | Laminin subunit alpha-5 OS=Homo sapiens OX=9606 GN=LAMA5 PE=1 SV=8 |
| P20908 | Collagen alpha-1(V) chain OS=Homo sapiens OX=9606 GN=COL5A1 PE=1 SV=3 |
| P98160 | Basement membrane-specific heparan sulfate proteoglycan core protein OS=Homo sapiens OX=9606 GN=HSPG2 PE=1 SV=4 |

| Degradation of the extracellular matrix | |
| --- | --- |
| P02462 | Collagen alpha-1(IV) chain OS=Homo sapiens OX=9606 GN=COL4A1 PE=1 SV=4 |
| P02461 | Collagen alpha-1(III) chain OS=Homo sapiens OX=9606 GN=COL3A1 PE=1 SV=4 |
| P05997 | Collagen alpha-2(V) chain OS=Homo sapiens OX=9606 GN=COL5A2 PE=1 SV=3 |
| O15230 | Laminin subunit alpha-5 OS=Homo sapiens OX=9606 GN=LAMA5 PE=1 SV=8 |
| P20908 | Collagen alpha-1(V) chain OS=Homo sapiens OX=9606 GN=COL5A1 PE=1 SV=3 |
| P98160 | Basement membrane-specific heparan sulfate proteoglycan core protein OS=Homo sapiens OX=9606 GN=HSPG2 PE=1 SV=4 |
| P13497 | Bone morphogenetic protein 1 OS=Homo sapiens OX=9606 GN=BMP1 PE=1 SV=2 |
| P14543 | Nidogen-1 OS=Homo sapiens OX=9606 GN=NID1 PE=1 SV=3 |

| Laminin interactions | |
| --- | --- |
| P02462 | Collagen alpha-1(IV) chain OS=Homo sapiens OX=9606 GN=COL4A1 PE=1 SV=4 |
| P55268 | Laminin subunit beta-2 OS=Homo sapiens OX=9606 GN=LAMB2 PE=1 SV=2 |
| O15230 | Laminin subunit alpha-5 OS=Homo sapiens OX=9606 GN=LAMA5 PE=1 SV=8 |
| P98160 | Basement membrane-specific heparan sulfate proteoglycan core protein OS=Homo sapiens OX=9606 GN=HSPG2 PE=1 SV=4 |
| P14543 | Nidogen-1 OS=Homo sapiens OX=9606 GN=NID1 PE=1 SV=3 |

| MET activates PTK2 signaling | |
| --- | --- |
| P02461 | Collagen alpha-1(III) chain OS=Homo sapiens OX=9606 GN=COL3A1 PE=1 SV=4 |
| P55268 | Laminin subunit beta-2 OS=Homo sapiens OX=9606 GN=LAMB2 PE=1 SV=2 |
| P05997 | Collagen alpha-2(V) chain OS=Homo sapiens OX=9606 GN=COL5A2 PE=1 SV=3 |
| O15230 | Laminin subunit alpha-5 OS=Homo sapiens OX=9606 GN=LAMA5 PE=1 SV=8 |
| P20908 | Collagen alpha-1(V) chain OS=Homo sapiens OX=9606 GN=COL5A1 PE=1 SV=3 |

| MET promotes cell motility | |
| --- | --- |
| P02461 | Collagen alpha-1(III) chain OS=Homo sapiens OX=9606 GN=COL3A1 PE=1 SV=4 |
| P55268 | Laminin subunit beta-2 OS=Homo sapiens OX=9606 GN=LAMB2 PE=1 SV=2 |
| P05997 | Collagen alpha-2(V) chain OS=Homo sapiens OX=9606 GN=COL5A2 PE=1 SV=3 |
| O15230 | Laminin subunit alpha-5 OS=Homo sapiens OX=9606 GN=LAMA5 PE=1 SV=8 |
| P20908 | Collagen alpha-1(V) chain OS=Homo sapiens OX=9606 GN=COL5A1 PE=1 SV=3 |

| Post-translational protein phosphorylation | |
| --- | --- |
| P55268 | Laminin subunit beta-2 OS=Homo sapiens OX=9606 GN=LAMB2 PE=1 SV=2 |
| P22692 | Insulin-like growth factor-binding protein 4 OS=Homo sapiens OX=9606 GN=IGFBP4 PE=1 SV=2 |
| P19022 | Cadherin-2 OS=Homo sapiens OX=9606 GN=CDH2 PE=1 SV=4 |
| Q14393 | Growth arrest-specific protein 6 OS=Homo sapiens OX=9606 GN=GAS6 PE=1 SV=3 |
| O95084 | Serine protease 23 OS=Homo sapiens OX=9606 GN=PRSS23 PE=1 SV=1 |
| O00622 | CCN family member 1 OS=Homo sapiens OX=9606 GN=CCN1 PE=1 SV=1 |

| Assembly of collagen fibrils and other multimeric structures | |
| --- | --- |
| P02462 | Collagen alpha-1(IV) chain OS=Homo sapiens OX=9606 GN=COL4A1 PE=1 SV=4 |
| P02461 | Collagen alpha-1(III) chain OS=Homo sapiens OX=9606 GN=COL3A1 PE=1 SV=4 |
| P05997 | Collagen alpha-2(V) chain OS=Homo sapiens OX=9606 GN=COL5A2 PE=1 SV=3 |
| P20908 | Collagen alpha-1(V) chain OS=Homo sapiens OX=9606 GN=COL5A1 PE=1 SV=3 |
| P13497 | Bone morphogenetic protein 1 OS=Homo sapiens OX=9606 GN=BMP1 PE=1 SV=2 |

| Collagen biosynthesis and modifying enzymes | |
| --- | --- |
| P02462 | Collagen alpha-1(IV) chain OS=Homo sapiens OX=9606 GN=COL4A1 PE=1 SV=4 |
| P02461 | Collagen alpha-1(III) chain OS=Homo sapiens OX=9606 GN=COL3A1 PE=1 SV=4 |
| P05997 | Collagen alpha-2(V) chain OS=Homo sapiens OX=9606 GN=COL5A2 PE=1 SV=3 |
| P20908 | Collagen alpha-1(V) chain OS=Homo sapiens OX=9606 GN=COL5A1 PE=1 SV=3 |
| P13497 | Bone morphogenetic protein 1 OS=Homo sapiens OX=9606 GN=BMP1 PE=1 SV=2 |

| Regulation of Insulin-like Growth Factor (IGF) transport and uptake by Insulin-like Growth Factor Binding Proteins (IGFBPs) | |
| --- | --- |
| P55268 | Laminin subunit beta-2 OS=Homo sapiens OX=9606 GN=LAMB2 PE=1 SV=2 |
| P22692 | Insulin-like growth factor-binding protein 4 OS=Homo sapiens OX=9606 GN=IGFBP4 PE=1 SV=2 |
| P19022 | Cadherin-2 OS=Homo sapiens OX=9606 GN=CDH2 PE=1 SV=4 |
| Q14393 | Growth arrest-specific protein 6 OS=Homo sapiens OX=9606 GN=GAS6 PE=1 SV=3 |
| O95084 | Serine protease 23 OS=Homo sapiens OX=9606 GN=PRSS23 PE=1 SV=1 |
| O00622 | CCN family member 1 OS=Homo sapiens OX=9606 GN=CCN1 PE=1 SV=1 |

| Signaling by MET | |
| --- | --- |
| P02461 | Collagen alpha-1(III) chain OS=Homo sapiens OX=9606 GN=COL3A1 PE=1 SV=4 |
| P55268 | Laminin subunit beta-2 OS=Homo sapiens OX=9606 GN=LAMB2 PE=1 SV=2 |
| P05997 | Collagen alpha-2(V) chain OS=Homo sapiens OX=9606 GN=COL5A2 PE=1 SV=3 |
| O15230 | Laminin subunit alpha-5 OS=Homo sapiens OX=9606 GN=LAMA5 PE=1 SV=8 |
| P20908 | Collagen alpha-1(V) chain OS=Homo sapiens OX=9606 GN=COL5A1 PE=1 SV=3 |

| Integrin cell surface interactions | |
| --- | --- |
| P02462 | Collagen alpha-1(IV) chain OS=Homo sapiens OX=9606 GN=COL4A1 PE=1 SV=4 |
| P02461 | Collagen alpha-1(III) chain OS=Homo sapiens OX=9606 GN=COL3A1 PE=1 SV=4 |
| P05997 | Collagen alpha-2(V) chain OS=Homo sapiens OX=9606 GN=COL5A2 PE=1 SV=3 |
| P20908 | Collagen alpha-1(V) chain OS=Homo sapiens OX=9606 GN=COL5A1 PE=1 SV=3 |
| P98160 | Basement membrane-specific heparan sulfate proteoglycan core protein OS=Homo sapiens OX=9606 GN=HSPG2 PE=1 SV=4 |

| NCAM1 interactions | |
| --- | --- |
| P02462 | Collagen alpha-1(IV) chain OS=Homo sapiens OX=9606 GN=COL4A1 PE=1 SV=4 |
| P02461 | Collagen alpha-1(III) chain OS=Homo sapiens OX=9606 GN=COL3A1 PE=1 SV=4 |
| P05997 | Collagen alpha-2(V) chain OS=Homo sapiens OX=9606 GN=COL5A2 PE=1 SV=3 |
| P20908 | Collagen alpha-1(V) chain OS=Homo sapiens OX=9606 GN=COL5A1 PE=1 SV=3 |

| Collagen formation | |
| --- | --- |
| P02462 | Collagen alpha-1(IV) chain OS=Homo sapiens OX=9606 GN=COL4A1 PE=1 SV=4 |
| P02461 | Collagen alpha-1(III) chain OS=Homo sapiens OX=9606 GN=COL3A1 PE=1 SV=4 |
| P05997 | Collagen alpha-2(V) chain OS=Homo sapiens OX=9606 GN=COL5A2 PE=1 SV=3 |
| P20908 | Collagen alpha-1(V) chain OS=Homo sapiens OX=9606 GN=COL5A1 PE=1 SV=3 |
| P13497 | Bone morphogenetic protein 1 OS=Homo sapiens OX=9606 GN=BMP1 PE=1 SV=2 |

**First 15 pathways based on 47 Down-regulated proteins**

| Pathway identifier | Pathway name | #Entities found | #Entities total | Entities pValue | Entities FDR |
| --- | --- | --- | --- | --- | --- |
| R-HSA-114608 | Platelet degranulation | 10 | 128 | 1.80E-10 | 3.83E-08 |
| R-HSA-76005 | Response to elevated platelet cytosolic Ca2+ | 10 | 133 | 2.60E-10 | 3.83E-08 |
| R-HSA-6798695 | Neutrophil degranulation | 15 | 478 | 9.29E-10 | 9.10E-08 |
| R-HSA-76002 | Platelet activation, signaling and aggregation | 11 | 265 | 1.42E-08 | 1.04E-06 |
| R-HSA-168256 | Immune System | 26 | 2188 | 1.42E-07 | 8.23E-06 |
| R-HSA-70171 | Glycolysis | 6 | 80 | 1.30E-06 | 6.39E-05 |
| R-HSA-9020591 | Interleukin-12 signaling | 5 | 46 | 1.77E-06 | 7.45E-05 |
| R-HSA-5628897 | TP53 Regulates Metabolic Genes | 6 | 88 | 2.25E-06 | 8.10E-05 |
| R-HSA-447115 | Interleukin-12 family signaling | 5 | 56 | 4.59E-06 | 1.35E-04 |
| R-HSA-70326 | Glucose metabolism | 6 | 100 | 4.66E-06 | 1.35E-04 |
| R-HSA-381183 | ATF6 (ATF6-alpha) activates chaperone genes | 3 | 10 | 1.21E-05 | 3.15E-04 |
| R-HSA-70263 | Gluconeogenesis | 4 | 35 | 1.70E-05 | 4.07E-04 |
| R-HSA-381033 | ATF6 (ATF6-alpha) activates chaperones | 3 | 12 | 2.08E-05 | 4.42E-04 |
| R-HSA-8950505 | Gene and protein expression by JAK-STAT signaling after Interleukin-12 stimulation | 4 | 37 | 2.10E-05 | 4.42E-04 |
| R-HSA-168249 | Innate Immune System | 16 | 1197 | 2.52E-05 | 4.48E-04 |

| Platelet degranulation | |
| --- | --- |
| P18206 | Vinculin OS=Homo sapiens OX=9606 GN=VCL PE=1 SV=4 |
| P11021 | Endoplasmic reticulum chaperone BiP OS=Homo sapiens OX=9606 GN=HSPA5 PE=1 SV=2 |
| P62937 | Peptidyl-prolyl cis-trans isomerase A OS=Homo sapiens OX=9606 GN=PPIA PE=1 SV=2 |
| P07737 | Profilin-1 OS=Homo sapiens OX=9606 GN=PFN1 PE=1 SV=2 |
| P12814 | Alpha-actinin-1 OS=Homo sapiens OX=9606 GN=ACTN1 PE=1 SV=2 |
| P62328 | Thymosin beta-4 OS=Homo sapiens OX=9606 GN=TMSB4X PE=1 SV=2 |
| P37802 | Transgelin-2 OS=Homo sapiens OX=9606 GN=TAGLN2 PE=1 SV=3 |
| P04075 | Fructose-bisphosphate aldolase A OS=Homo sapiens OX=9606 GN=ALDOA PE=1 SV=2 |
| P21333 | Filamin-A OS=Homo sapiens OX=9606 GN=FLNA PE=1 SV=4 |
| Q9Y490 | Talin-1 OS=Homo sapiens OX=9606 GN=TLN1 PE=1 SV=3 |

| Response to elevated platelet cytosolic Ca2+ | |
| --- | --- |
| P18206 | Vinculin OS=Homo sapiens OX=9606 GN=VCL PE=1 SV=4 |
| P11021 | Endoplasmic reticulum chaperone BiP OS=Homo sapiens OX=9606 GN=HSPA5 PE=1 SV=2 |
| P62937 | Peptidyl-prolyl cis-trans isomerase A OS=Homo sapiens OX=9606 GN=PPIA PE=1 SV=2 |
| P07737 | Profilin-1 OS=Homo sapiens OX=9606 GN=PFN1 PE=1 SV=2 |
| P12814 | Alpha-actinin-1 OS=Homo sapiens OX=9606 GN=ACTN1 PE=1 SV=2 |
| P62328 | Thymosin beta-4 OS=Homo sapiens OX=9606 GN=TMSB4X PE=1 SV=2 |
| P37802 | Transgelin-2 OS=Homo sapiens OX=9606 GN=TAGLN2 PE=1 SV=3 |
| P04075 | Fructose-bisphosphate aldolase A OS=Homo sapiens OX=9606 GN=ALDOA PE=1 SV=2 |
| P21333 | Filamin-A OS=Homo sapiens OX=9606 GN=FLNA PE=1 SV=4 |
| Q9Y490 | Talin-1 OS=Homo sapiens OX=9606 GN=TLN1 PE=1 SV=3 |

| Neutrophil degranulation | |
| --- | --- |
| P06744 | Glucose-6-phosphate isomerase OS=Homo sapiens OX=9606 GN=GPI PE=1 SV=4 |
| P50395 | Rab GDP dissociation inhibitor beta OS=Homo sapiens OX=9606 GN=GDI2 PE=1 SV=2 |
| P14618 | Pyruvate kinase PKM OS=Homo sapiens OX=9606 GN=PKM PE=1 SV=4 |
| P62937 | Peptidyl-prolyl cis-trans isomerase A OS=Homo sapiens OX=9606 GN=PPIA PE=1 SV=2 |
| P18206 | Vinculin OS=Homo sapiens OX=9606 GN=VCL PE=1 SV=4 |
| P11142 | Heat shock cognate 71 kDa protein OS=Homo sapiens OX=9606 GN=HSPA8 PE=1 SV=1 |
| P36871 | Phosphoglucomutase-1 OS=Homo sapiens OX=9606 GN=PGM1 PE=1 SV=3 |
| P68104 | Elongation factor 1-alpha 1 OS=Homo sapiens OX=9606 GN=EEF1A1 PE=1 SV=1 |
| Q13162 | Peroxiredoxin-4 OS=Homo sapiens OX=9606 GN=PRDX4 PE=1 SV=1 |
| P31949 | Protein S100-A11 OS=Homo sapiens OX=9606 GN=S100A11 PE=1 SV=2 |
| P04075 | Fructose-bisphosphate aldolase A OS=Homo sapiens OX=9606 GN=ALDOA PE=1 SV=2 |
| P09211 | Glutathione S-transferase P OS=Homo sapiens OX=9606 GN=GSTP1 PE=1 SV=2 |
| P04264 | Keratin, type II cytoskeletal 1 OS=Homo sapiens OX=9606 GN=KRT1 PE=1 SV=6 |
| P07355 | Annexin A2 OS=Homo sapiens OX=9606 GN=ANXA2 PE=1 SV=2 |
| Q92820 | Gamma-glutamyl hydrolase OS=Homo sapiens OX=9606 GN=GGH PE=1 SV=2 |

| Platelet activation, signaling and aggregation | |
| --- | --- |
| P18206 | Vinculin OS=Homo sapiens OX=9606 GN=VCL PE=1 SV=4 |
| P11021 | Endoplasmic reticulum chaperone BiP OS=Homo sapiens OX=9606 GN=HSPA5 PE=1 SV=2 |
| P62937 | Peptidyl-prolyl cis-trans isomerase A OS=Homo sapiens OX=9606 GN=PPIA PE=1 SV=2 |
| P07737 | Profilin-1 OS=Homo sapiens OX=9606 GN=PFN1 PE=1 SV=2 |
| P12814 | Alpha-actinin-1 OS=Homo sapiens OX=9606 GN=ACTN1 PE=1 SV=2 |
| P62328 | Thymosin beta-4 OS=Homo sapiens OX=9606 GN=TMSB4X PE=1 SV=2 |
| P04075 | Fructose-bisphosphate aldolase A OS=Homo sapiens OX=9606 GN=ALDOA PE=1 SV=2 |
| P37802 | Transgelin-2 OS=Homo sapiens OX=9606 GN=TAGLN2 PE=1 SV=3 |
| P63104 | 14-3-3 protein zeta/delta OS=Homo sapiens OX=9606 GN=YWHAZ PE=1 SV=1 |
| Q9Y490 | Talin-1 OS=Homo sapiens OX=9606 GN=TLN1 PE=1 SV=3 |
| P21333 | Filamin-A OS=Homo sapiens OX=9606 GN=FLNA PE=1 SV=4 |

| Immune System | |
| --- | --- |
| P50395 | Rab GDP dissociation inhibitor beta OS=Homo sapiens OX=9606 GN=GDI2 PE=1 SV=2 |
| P14618 | Pyruvate kinase PKM OS=Homo sapiens OX=9606 GN=PKM PE=1 SV=4 |
| P62937 | Peptidyl-prolyl cis-trans isomerase A OS=Homo sapiens OX=9606 GN=PPIA PE=1 SV=2 |
| O75369 | Filamin-B OS=Homo sapiens OX=9606 GN=FLNB PE=1 SV=2 |
| P30101 | Protein disulfide-isomerase A3 OS=Homo sapiens OX=9606 GN=PDIA3 PE=1 SV=4 |
| P36871 | Phosphoglucomutase-1 OS=Homo sapiens OX=9606 GN=PGM1 PE=1 SV=3 |
| Q13162 | Peroxiredoxin-4 OS=Homo sapiens OX=9606 GN=PRDX4 PE=1 SV=1 |
| P09211 | Glutathione S-transferase P OS=Homo sapiens OX=9606 GN=GSTP1 PE=1 SV=2 |
| P04264 | Keratin, type II cytoskeletal 1 OS=Homo sapiens OX=9606 GN=KRT1 PE=1 SV=6 |
| P26038 | Moesin OS=Homo sapiens OX=9606 GN=MSN PE=1 SV=3 |
| P63104 | 14-3-3 protein zeta/delta OS=Homo sapiens OX=9606 GN=YWHAZ PE=1 SV=1 |
| P07355 | Annexin A2 OS=Homo sapiens OX=9606 GN=ANXA2 PE=1 SV=2 |
| P07237 | Protein disulfide-isomerase OS=Homo sapiens OX=9606 GN=P4HB PE=1 SV=3 |
| P06744 | Glucose-6-phosphate isomerase OS=Homo sapiens OX=9606 GN=GPI PE=1 SV=4 |
| P14625 | Endoplasmin OS=Homo sapiens OX=9606 GN=HSP90B1 PE=1 SV=1 |
| P18206 | Vinculin OS=Homo sapiens OX=9606 GN=VCL PE=1 SV=4 |
| P22626 | Heterogeneous nuclear ribonucleoproteins A2/B1 OS=Homo sapiens OX=9606 GN=HNRNPA2B1 PE=1 SV=2 |
| P11142 | Heat shock cognate 71 kDa protein OS=Homo sapiens OX=9606 GN=HSPA8 PE=1 SV=1 |
| P11021 | Endoplasmic reticulum chaperone BiP OS=Homo sapiens OX=9606 GN=HSPA5 PE=1 SV=2 |
| P68104 | Elongation factor 1-alpha 1 OS=Homo sapiens OX=9606 GN=EEF1A1 PE=1 SV=1 |
| P08670 | Vimentin OS=Homo sapiens OX=9606 GN=VIM PE=1 SV=4 |
| P31949 | Protein S100-A11 OS=Homo sapiens OX=9606 GN=S100A11 PE=1 SV=2 |
| P04075 | Fructose-bisphosphate aldolase A OS=Homo sapiens OX=9606 GN=ALDOA PE=1 SV=2 |
| P27797 | Calreticulin OS=Homo sapiens OX=9606 GN=CALR PE=1 SV=1 |
| Q92820 | Gamma-glutamyl hydrolase OS=Homo sapiens OX=9606 GN=GGH PE=1 SV=2 |
| P21333 | Filamin-A OS=Homo sapiens OX=9606 GN=FLNA PE=1 SV=4 |

| Glycolysis | |
| --- | --- |
| P06733 | Alpha-enolase OS=Homo sapiens OX=9606 GN=ENO1 PE=1 SV=2 |
| P06744 | Glucose-6-phosphate isomerase OS=Homo sapiens OX=9606 GN=GPI PE=1 SV=4 |
| P14618 | Pyruvate kinase PKM OS=Homo sapiens OX=9606 GN=PKM PE=1 SV=4 |
| P00558 | Phosphoglycerate kinase 1 OS=Homo sapiens OX=9606 GN=PGK1 PE=1 SV=3 |
| P04075 | Fructose-bisphosphate aldolase A OS=Homo sapiens OX=9606 GN=ALDOA PE=1 SV=2 |

| Interleukin-12 signaling | |
| --- | --- |
| P22626 | Heterogeneous nuclear ribonucleoproteins A2/B1 OS=Homo sapiens OX=9606 GN=HNRNPA2B1 PE=1 SV=2 |
| P62937 | Peptidyl-prolyl cis-trans isomerase A OS=Homo sapiens OX=9606 GN=PPIA PE=1 SV=2 |
| P26038 | Moesin OS=Homo sapiens OX=9606 GN=MSN PE=1 SV=3 |
| P07355 | Annexin A2 OS=Homo sapiens OX=9606 GN=ANXA2 PE=1 SV=2 |
| P07237 | Protein disulfide-isomerase OS=Homo sapiens OX=9606 GN=P4HB PE=1 SV=3 |

| TP53 Regulates Metabolic Genes | |
| --- | --- |
| P06744 | Glucose-6-phosphate isomerase OS=Homo sapiens OX=9606 GN=GPI PE=1 SV=4 |
| Q06830 | Peroxiredoxin-1 OS=Homo sapiens OX=9606 GN=PRDX1 PE=1 SV=1 |
| P27348 | 14-3-3 protein theta OS=Homo sapiens OX=9606 GN=YWHAQ PE=1 SV=1 |
| P63104 | 14-3-3 protein zeta/delta OS=Homo sapiens OX=9606 GN=YWHAZ PE=1 SV=1 |
| P62258 | 14-3-3 protein epsilon OS=Homo sapiens OX=9606 GN=YWHAE PE=1 SV=1 |
| Q16881 | Thioredoxin reductase 1, cytoplasmic OS=Homo sapiens OX=9606 GN=TXNRD1 PE=1 SV=3 |

| Interleukin-12 family signaling | |
| --- | --- |
| P22626 | Heterogeneous nuclear ribonucleoproteins A2/B1 OS=Homo sapiens OX=9606 GN=HNRNPA2B1 PE=1 SV=2 |
| P62937 | Peptidyl-prolyl cis-trans isomerase A OS=Homo sapiens OX=9606 GN=PPIA PE=1 SV=2 |
| P26038 | Moesin OS=Homo sapiens OX=9606 GN=MSN PE=1 SV=3 |
| P07355 | Annexin A2 OS=Homo sapiens OX=9606 GN=ANXA2 PE=1 SV=2 |
| P07237 | Protein disulfide-isomerase OS=Homo sapiens OX=9606 GN=P4HB PE=1 SV=3 |

| Glucose metabolism | |
| --- | --- |
| P06733 | Alpha-enolase OS=Homo sapiens OX=9606 GN=ENO1 PE=1 SV=2 |
| P06744 | Glucose-6-phosphate isomerase OS=Homo sapiens OX=9606 GN=GPI PE=1 SV=4 |
| P14618 | Pyruvate kinase PKM OS=Homo sapiens OX=9606 GN=PKM PE=1 SV=4 |
| P00558 | Phosphoglycerate kinase 1 OS=Homo sapiens OX=9606 GN=PGK1 PE=1 SV=3 |
| P04075 | Fructose-bisphosphate aldolase A OS=Homo sapiens OX=9606 GN=ALDOA PE=1 SV=2 |

| ATF6 (ATF6-alpha) activates chaperone genes | |
| --- | --- |
| P11021 | Endoplasmic reticulum chaperone BiP OS=Homo sapiens OX=9606 GN=HSPA5 PE=1 SV=2 |
| P14625 | Endoplasmin OS=Homo sapiens OX=9606 GN=HSP90B1 PE=1 SV=1 |
| P27797 | Calreticulin OS=Homo sapiens OX=9606 GN=CALR PE=1 SV=1 |

| Gluconeogenesis | |
| --- | --- |
| P06733 | Alpha-enolase OS=Homo sapiens OX=9606 GN=ENO1 PE=1 SV=2 |
| P06744 | Glucose-6-phosphate isomerase OS=Homo sapiens OX=9606 GN=GPI PE=1 SV=4 |
| P00558 | Phosphoglycerate kinase 1 OS=Homo sapiens OX=9606 GN=PGK1 PE=1 SV=3 |
| P04075 | Fructose-bisphosphate aldolase A OS=Homo sapiens OX=9606 GN=ALDOA PE=1 SV=2 |

| ATF6 (ATF6-alpha) activates chaperones | |
| --- | --- |
| P11021 | Endoplasmic reticulum chaperone BiP OS=Homo sapiens OX=9606 GN=HSPA5 PE=1 SV=2 |
| P14625 | Endoplasmin OS=Homo sapiens OX=9606 GN=HSP90B1 PE=1 SV=1 |
| P27797 | Calreticulin OS=Homo sapiens OX=9606 GN=CALR PE=1 SV=1 |

| Gene and protein expression by JAK-STAT signaling after Interleukin-12 stimulation | |
| --- | --- |
| P22626 | Heterogeneous nuclear ribonucleoproteins A2/B1 OS=Homo sapiens OX=9606 GN=HNRNPA2B1 PE=1 SV=2 |
| P62937 | Peptidyl-prolyl cis-trans isomerase A OS=Homo sapiens OX=9606 GN=PPIA PE=1 SV=2 |
| P26038 | Moesin OS=Homo sapiens OX=9606 GN=MSN PE=1 SV=3 |
| P07355 | Annexin A2 OS=Homo sapiens OX=9606 GN=ANXA2 PE=1 SV=2 |

| Innate Immune System | |
| --- | --- |
| P06744 | Glucose-6-phosphate isomerase OS=Homo sapiens OX=9606 GN=GPI PE=1 SV=4 |
| P50395 | Rab GDP dissociation inhibitor beta OS=Homo sapiens OX=9606 GN=GDI2 PE=1 SV=2 |
| P14618 | Pyruvate kinase PKM OS=Homo sapiens OX=9606 GN=PKM PE=1 SV=4 |
| P62937 | Peptidyl-prolyl cis-trans isomerase A OS=Homo sapiens OX=9606 GN=PPIA PE=1 SV=2 |
| P14625 | Endoplasmin OS=Homo sapiens OX=9606 GN=HSP90B1 PE=1 SV=1 |
| P18206 | Vinculin OS=Homo sapiens OX=9606 GN=VCL PE=1 SV=4 |
| P11142 | Heat shock cognate 71 kDa protein OS=Homo sapiens OX=9606 GN=HSPA8 PE=1 SV=1 |
| P36871 | Phosphoglucomutase-1 OS=Homo sapiens OX=9606 GN=PGM1 PE=1 SV=3 |
| P68104 | Elongation factor 1-alpha 1 OS=Homo sapiens OX=9606 GN=EEF1A1 PE=1 SV=1 |
| Q13162 | Peroxiredoxin-4 OS=Homo sapiens OX=9606 GN=PRDX4 PE=1 SV=1 |
| P31949 | Protein S100-A11 OS=Homo sapiens OX=9606 GN=S100A11 PE=1 SV=2 |
| P04075 | Fructose-bisphosphate aldolase A OS=Homo sapiens OX=9606 GN=ALDOA PE=1 SV=2 |
| P09211 | Glutathione S-transferase P OS=Homo sapiens OX=9606 GN=GSTP1 PE=1 SV=2 |
| P04264 | Keratin, type II cytoskeletal 1 OS=Homo sapiens OX=9606 GN=KRT1 PE=1 SV=6 |
| P07355 | Annexin A2 OS=Homo sapiens OX=9606 GN=ANXA2 PE=1 SV=2 |
| Q92820 | Gamma-glutamyl hydrolase OS=Homo sapiens OX=9606 GN=GGH PE=1 SV=2 |
